# Depictability of the upper gastrointestinal tract on forward‐viewing radial endoscopic ultrasonography versus standard upper esophagogastroduodenoscopy

**DOI:** 10.1002/deo2.89

**Published:** 2022-01-24

**Authors:** Tesshin Ban, Yoshimasa Kubota, Takuya Takahama, Shun Sasoh, Tomoaki Ando, Makoto Nakamura, Takashi Joh

**Affiliations:** ^1^ Department of Gastroenterology and Hepatology Gamagori Municipal Hospital Aichi Japan

**Keywords:** esophagogastroduodenoscopy, forward‐viewing radial endoscopic ultrasonography, forward‐viewing radial EUS, upper gastrointestinal tract, upper GI

## Abstract

**Objectives:**

Esophagogastroduodenoscopy (EGD) is a widely used modality for investigating the upper gastrointestinal (GI) tract, similar to endoscopic ultrasonography (EUS) for the pancreaticobiliary system. A recent and novel forward‐viewing radial EUS has potential as an EGD. However, this potential has not yet been evaluated and reported in the literature. We compared the depictability of the upper GI tract on EUS using a standard EGD.

**Methods:**

This was a prospective study in a single session in an identical patient and it was conducted at a single center.

**Results:**

Sixty‐nine participants were enrolled in this study. A forward‐viewing radial EUS revealed equivalent visualizing performance compared with the standard EGD, except for the retroflex view of the three angular areas. Depiction scores of the anterior wall, lesser curvature, and posterior wall of the angulus in the retroflex view in the forward‐viewing radial EUS were 1.94 (95% confidence interval [CI], 1.36–2.52), 2.03 (95% CI, 1.48–2.58), and 1.93 (95% CI, 1.35–2.50), respectively. These scores were significantly lower compared with those of standard EGD scores of 2.97 (95% CI, 2.86–3.08), 2.97 (95% CI, 2.86–3.78), and 2.96 (95% CI, 2.83–3.09], respectively; *p* < 0.001). The rate of full‐mark score in these three angular areas was significantly lower in the forward‐viewing radial EUS than in the standard EGD (21/69, 30.4%; 23/69, 33.3%; 21/69, 30.4% vs. 67/69; 97.1%, 67/69; 97.1%, 66/69; 95.7%, *p *< 0.001 for all).

**Conclusions:**

Although forward‐viewing radial EUS has the potential to simultaneously investigate the upper GI and pancreaticobiliary systems, it is too early to introduce it for this purpose.

## INTRODUCTION

Esophagogastroduodenoscopy (EGD) is a widespread abdominal examination for upper abdominal symptoms (e.g., pain, anorexia, weight loss, dysphagia, vomiting, and acid reflux), similar to endoscopic ultrasonography for the evaluation of abnormalities of the upper gastrointestinal (GI) tract wall or adjacent structures including the pancreaticobiliary system.^1^ Ideally, the GI tract and pancreaticobiliary system are investigated in a single session using single endoscopic ultrasonography (EUS). This concept suggests that endoscopists cover the entire upper GI tract and pancreaticobiliary system with a single scope, except for the liver and intrahepatic bile duct. Previous studies have described this possibility using radial EUS.^2^, ^3^ However, a most recent study revealed that the blind spot area of 26 sections in the GI tract was 22.46%, even with standard EGD.^4^ A recent endoscopic ultrasonic processor and forward‐viewing radial EUS potentially have the simultaneous performance of EGD and EUS.^3^ However, this advantage has not been investigated well in the sectionalized upper GI tract.

This prospective diagnostic study aimed to evaluate the depictability of the upper GI tract on cutting‐edge forward‐viewing radial EUS in comparison with the standard EGD.

## METHODS

### Study design

This study was a prospective study to evaluate the depictability of the upper GI tract on forward‐viewing radial EUS compared with standard EGD in a single session and an identical patient.

### Study population

After protocol approval by the Institutional Review Board of Gamagori Municipal Hospital, we recruited consecutive patients who visited our hospital with upper abdominal symptoms. Inclusion criteria were as follows: (1) scheduled for both standard EGD and EUS, (2) age ≥20 years, and (3) provision of voluntary written consent for participation in this study. The exclusion criteria were as follows: (1) age less than 20 years, (2) surgically altered upper GI anatomy, (3) poor grade of the American Society of Anesthesiologists physical status^5^ with III or more, (4) prothrombin time‐international normalized ratio >3.0, and (5) ineligibility for inclusion as judged by the attending physician.

### Examination methods

Endoscopic examinations were carried out with the patient in the left lateral decubitus position under moderate sedation using benzodiazepines (midazolam, 0.03 mg/kg) plus opioid receptor agonists (pentazocine, 15.0 mg/body) administered intravenously.^6^, ^7^, ^8^ Additional midazolam (1.0 mg) was injected appropriately to maintain a Ramsey score of 3 or 4.^9^

**FIGURE 1:**
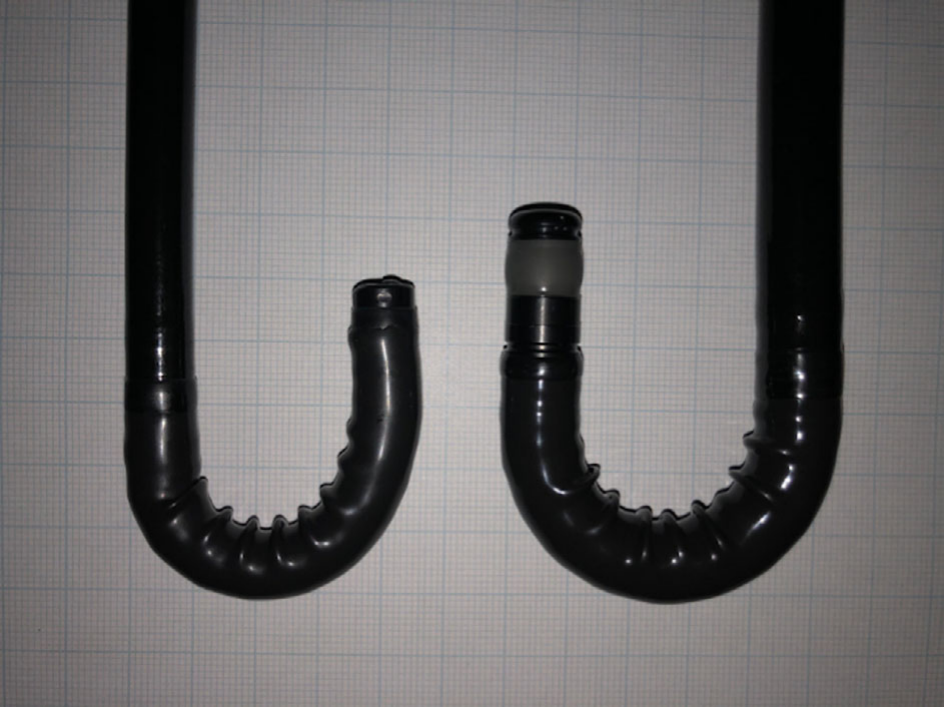
Photographs of standard EGD and forward‐viewing radial EUS bent at 180° upward. Left, standard upper EGD (EG‐L 600 WR7; Fujifilm, Tokyo, Japan). Right, forward‐viewing radial EUS (EG‐580UR; Fujifilm). EGD, esophagogastroduodenoscopy; EUS, endoscopic ultrasonography

A standard EGD (EG‐L 600 WR7; Fujifilm, Tokyo, Japan) was used as a reference. We consecutively captured upper GI images according to a previous study.^10^ No data exists to support evidence‐based photodocumentation of all typical anatomical landmarks.^11^ The minimum number of pictures to be collected in a routine endoscopic examination should be 10, namely, the proximal esophagus, distal esophagus, Z line and diaphragm indentation, cardia and fundus in inversion, corpus in forward view (including lesser curvature), corpus in retroflex view (including the greater curvature), angulus in partial inversion, antrum, duodenal bulb, and the second part of the duodenum.^11^ In this study, in line with a previous study,^4^ the captured upper GI images were classified into the following 26 sections: area 1, esophagus; area 2, esophagogastric junction; area 3, anterior wall of antrum; area 4, posterior wall of antrum; area 5, lesser curvature of antrum; area 6, greater curvature of antrum; area 7, anterior wall of lower body; area 8, posterior wall of lower body; area 9, lesser curvature of lower body; area 10, greater curvature of lower body; area 11, anterior wall of middle body; area 12, posterior wall of middle body; area 13, lesser curvature of middle body; area 14, greater curvature of middle body; area 15, anterior wall of middle body in retroflex view; area 16, posterior wall of middle body in retroflex view; area 17, lesser curvature of middle body in retroflex view; area 18, anterior wall of fundus in retroflex view; area 19, posterior wall of fundus in retroflex view; area 20, lesser curvature of fundus in retroflex view; area 21, greater curvature of fundus in retroflex view; area 22, anterior wall of angulus in retroflex view; area 23, lesser curvature of angulus in retroflex view; area 24, posterior wall of angulus in retroflex view; area 25, duodenal bulb; and area 26, descending duodenum. The index test was the endoscopic mode of forward‐viewing radial EUS. Forward‐viewing radial EUS (EG‐580UR; Fujifilm) with an endoscopic ultrasonic processor (SU1; Fujifilm) was performed accordingly. The specifications of both scopes are listed in Table 1. The actual length and width of the hard part of the scope tips bent at 180° upward (Figure 1) were measured thrice using a Vernier caliper, given that these specifications were not disclosed commercially. The order of scope intubation depended on the patient's symptoms and the endoscopist's discretion. Upper GI investigation and assessment in standard EGD and forward‐viewing radial EUS were performed by a single endoscopist, in a single session, and in an identical patient. Investigators Tesshin Ban and Yoshimasa Kubota were experts with a carrier of over 10,000 EGDs and over 1000 EUSs, and Takuya Takahama and Shun Sasoh had a carrier of over 1000 EGDs; however, they were trainees of EUS. When the first investigator was a trainee, the procedures were performed under the supervision of experts.

**TABLE 1:** Endoscopic specification of standard upper esophagogastroduodenoscopy (EGD) and forward‐viewing radial endoscopic ultrasonography (EUS)

			**EG‐L 600 WR7**	**EG‐580UR**
View direction			0°	0°
View angle			140°	140°
Scope diameter			9.2°	11.4°
Curving ability	up		210°	190°
	down		90°	90°
	right		100°	100°
	left		100°	100°
Hard part of the tip	length	mm (SD)	45.1 (0.1)	56.2 (0.1)
	width	mm (SD)	41.6 (0)	48.3 (0)

Abbreviations: EGD, esophagogastroduodenoscopy; EUS, endoscopic ultrasonography; SD, standard deviation.

### Outcome measures

**FIGURE 2:**
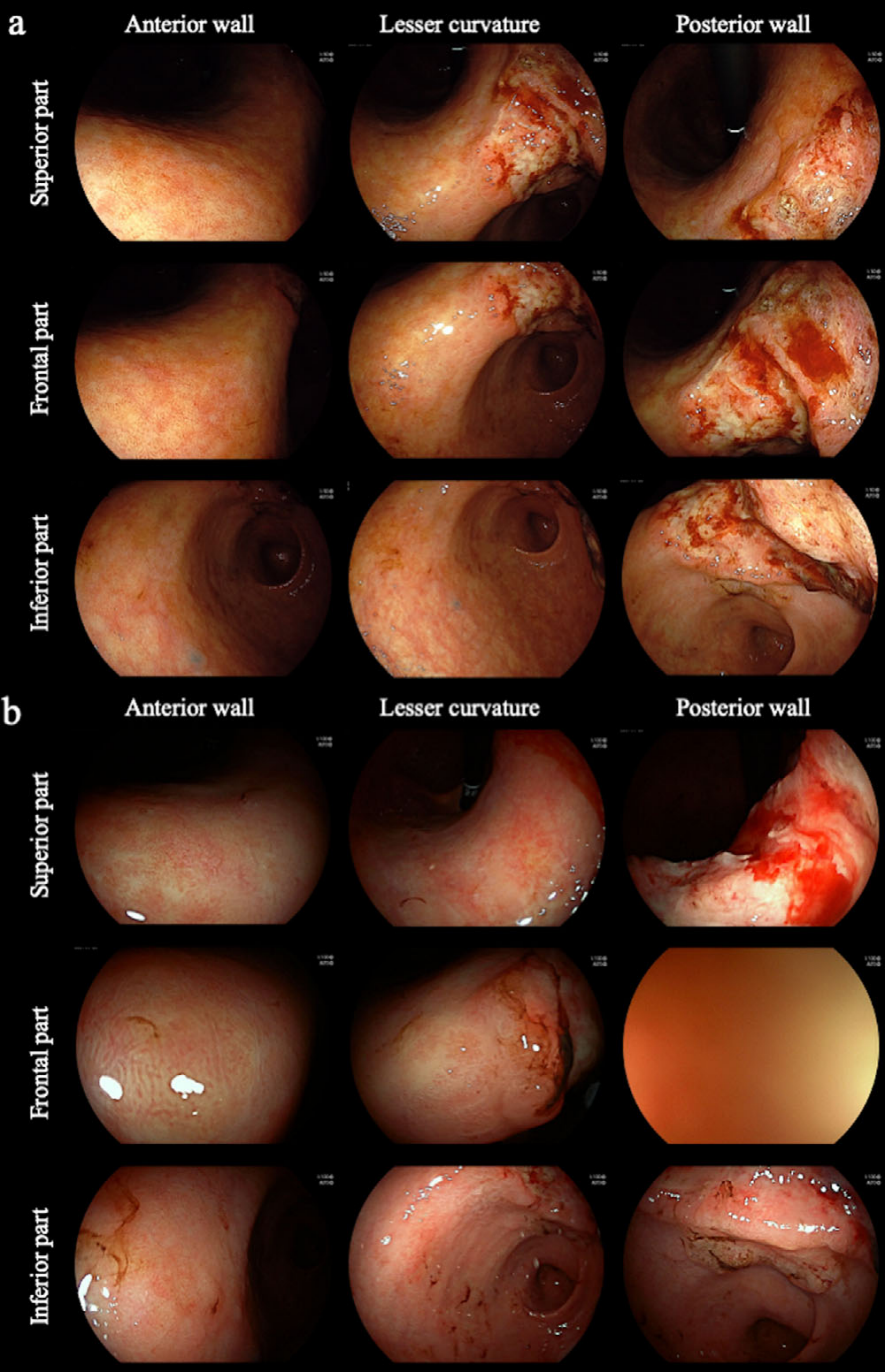
Representative captured endoscopic images at three angular areas in retroflex view using standard EGD and forward‐viewing radial EUS. Gastric cancer at the lesser curvature, posterior wall of the angulus in the retroflex view. (a) Endoscopic images captured using the standard EGD. Depictability scores were 3 in all areas of the anterior wall, lesser curvature, and posterior wall. (b) Captured endoscopic images using forward‐viewing radial EUS. Depictability scores were 3 in the anterior wall and lesser curvature; however, the score was 2 in the posterior wall. Left column, anterior wall; middle column, lesser curvature; right column, posterior wall; upper row, superior part; middle row, frontal part; and lower row, inferior part. EGD, esophagogastroduodenoscopy; EUS, endoscopic ultrasonography

The main outcome measure was the depictability of the 26 upper GI areas in both scopes. Originally, depictability was scored as follows: score 3 (full mark), able to depict mucosal structure, for example, vascular structure, in the whole area of interest; score 2, unable to depict mucosal structure in some areas of interest; score 1, unable to depict mucosal structure in almost the whole area of interest; and score 0, inaccessible to the area of interest. For investigation of the three angular areas, scoring was performed as follows: score 3 (full mark), able to depict mucosal structure in the whole area of interest (superior part, frontal part, and inferior part of the interest); score 2, unable to depict mucosal structure in one‐third of the area of interest; score 1, unable to depict mucosal structure in two‐thirds of the area of interest; and score 0, inaccessible to the area of interest (Figure 2).

The secondary outcome measures were the endoscopic gastric findings of both procedures. In particular, chronic gastritis, potential high‐risk endoscopic findings for gastric cancer, including atrophy (close‐0, 1, 2, 3, open‐1, 2, 3), intestinal metaplasia (absent, antrum, corpus), fold enlargement (absent, present), diffuse redness (absent, present), regular arrangement of collecting venules (visible, invisible), and map‐like redness (absent, antrum, corpus), were recorded with reference to a previous study based on the Kyoto classification.^12^ Adverse events were recorded according to the lexicon for endoscopic adverse events.^13^

In addition to the outcome measures described above, the following variables were prospectively recorded: chief complaint/purpose of this investigation, age, sex, endoscopist, order of scope intubation, number of upper GI images, duration, and total dosage of sedative‐analgesic agents.

### Statistical analysis

This was a single‐armed, observational, and feasibility study. The sample size was estimated using G*power 3.1 software (https://www.psychologie.hhu.de/arbeitsgruppen/allgemeine‐psychologie‐und‐arbeitspsychologie/gpower)^14^ as follows: crude sample size would be 64 participants with an effect size = 0.47, *α*‐error = 0.05, and *β*‐error = 0.95, using the two‐tailed Wilcoxon signed‐rank test. Approximately 10% of the participants were estimated to have dropped out for unavoidable reasons, such as missing data, protocol violation, and hemostasis. Therefore, the final sample size was set to 70.

The scores of depictability of 26 upper GI areas, number of endoscopic images, and duration of upper GI investigation in both scopes were compared using the Wilcoxon signed‐rank test with a 95% confidence interval (CI). The rate of full‐mark scores in both scopes was compared using McNemar's test.

Concordance rates of endoscopic findings of chronic gastritis in both scopes were described using the kappa statistics (κ) with 95% CI.

Statistical significance was set at *p* < 0.05. IBM SPSS Statistics 28 (IBM Japan Ltd., Tokyo, Japan) was used for all analyses.

### Ethics statement

All procedures followed were in accordance with the ethical standards of the responsible committee on human experimentation and conform to the provisions of the Declaration of Helsinki of 1964 and its later versions. Written informed consent was obtained from all patients. The protocol was approved by the Institutional Review Board of Gamagori Municipal Hospital (approval number: 614) and registered in the UMIN protocol (UMIN 000043487).

## RESULTS

### Characteristics of participants and investigations

Ongoing upper GI bleeding was found in one of the 70 participants in whom investigation was started with forward‐viewing radial EUS. Hence, a EUS scan was terminated in this case and hemostasis was performed accordingly. Therefore, the remaining 69 participants underwent both standard EGD and forward‐viewing radial EUS in a single session. The baseline characteristics of the participants and their investigations are summarized in Table 2. The median age was 72 years (range 24–93), and 32 participants (45.7%) were men. Thirty‐two participants (45.7%) presented with abdominal pain, and four participants (5.7%) presented with back pain. Other participants participated in this study for surveillance or diagnostic examinations. Forty‐four investigations (62.9%) were performed by experts, and the remaining were supervised by experts. Twenty‐eight investigations (40.0%) were started with forward‐viewing radial EUS followed by standard EGD. The number of captured upper GI images in standard EGD and forward‐viewing radial EUS, and the duration of observation were 59 and 61 images, and 329 and 324 s, respectively. The total dosages of sedative‐analgesic agents were 3.8 mg of midazolam and 15.0 mg of pentazocine.

**TABLE 2:** Baseline characteristics of participants and investigations, *N* = 69

Age	years	median (range)	72	(24–93)
Gender	male	number (%)	32	(45.7)
Chief complaint/purpose	abdominal pain	number (%)	32	(45.7)
	backpain		4	(5.7)
	surveillance		22	(31.4)
	OJ		6	(8.6)
	PB tumor		4	(5.7)
	elevated CA19‐9		1	(1.4)
Endoscopist	expert	number (%)	44	(62.9)
Order of scope intubation	EUS to EGD	number (%)	28	(40.0)
Captured upper GI images	standard EGD	number, median (range)	59	(32–111)
	EGD mode in EUS		61	(25–90)
Duration of observation	standard EGD	seconds, median (range)	329	(166–1061)
	EGD mode in EUS		324	(162–666)
	EUS		894	(231–2421)
	overall		1787	(975–3210)
Sedative‐analgesic agents	Midazolam	mg, median (range)	3.8	(1.2–6.8)
	Pentazocine		15.0	(15.0–15.0)

Abbreviations: CA19‐9, carbohydrate antigen 19‐9; EGD, esophagogastroduodenoscopy; EUS, endoscopic ultrasonography; GI, gastrointestinal tract; OJ, obstructive jaundice; PB tumor, pancreaticobiliary tumor.

### Depictability of 26 upper GI areas on both scopes

The depictions of the 26 upper GI areas on both scopes are summarized in Table 3. Forward‐viewing radial EUS revealed equivalent upper GI visualizing performance compared with standard EGD, except for the retroflex view for three angular areas. Scores expressed in mean in forward‐viewing radial EUS for the anterior wall, lesser curvature, and posterior wall of the angulus in the retroflex view were 1.94 (95% CI, 1.36–2.52), 2.03 (95% CI, 1.48–2.58), and 1.93 (95% CI, 1.35–2.50), respectively. These scores were significantly lower compared with those of standard EGD these being 2.97 (95% CI, 2.86–3.08), 2.97 (95% CI, 2.86–3.78), and 2.96 (95% CI, 2.83–3.09) respectively (*p* < 0.001). The rate of full‐mark score in the anterior wall, lesser curvature, and posterior wall of the angulus in the retroflex view were significantly lower in forward‐viewing radial EUS (21/69, 30.4%; 23/69, 33.3%; 21/69, 30.4% vs. 67/69, 97.1%; 67/69, 97.1%; 66/69, 95.7%; *p* < 0.001 for all). The rate of full‐mark score in all 26 areas in both scopes was significantly lower in forward‐viewing radial EUS (14/69, 20.3% vs. 66/69, 95.7%; *p* < .001).

**TABLE 3:** Depictability of 26 upper gastrointestinal tract areas on both scopes, *N* = 69

		**Standard EGD**		**95% CI**	**Forward‐viewing radial EUS**		**95% CI**	***p*‐value**
Angulus, ant. (J)	score, mean (range)	2.97	(2–3)	2.86–3.08	1.94	(0–3)	1.36–2.52	<0.001*
	full mark, n. (%)	67	97.1%		21	30.4%		<0.001*
Angulus, lesser (J)	score, mean (range)	2.97	(2–3)	2.86–3.78	2.03	(0–3)	1.48–2.58	<0.001*
	full mark, n. (%)	67	97.1%		23	33.3%		<0.001*
Angulus, post. (J)	score, mean (range)	2.96	(2–3)	2.83–3.09	1.93	(0‐3)	1.35–2.50	<0.001*
	full mark, *n* (%)	66	95.7%		21	30.4%		<0.001*
Cardia, greater (J)	score, mean (range)	2.99	(2–3)		2.99	(2–3)		1
Other 22 areas	score, mean (range)	3			3			1

Abbreviations: Angulus, ant. (J), anterior wall of angulus in retroflex view; Angulus, lesser (J), lesser curvature of angulus in retroflex view; Angulus, post. (J), posterior wall of angulus in retroflex view; Cardia, greater (J), greater curvature of the fundus in retroflex view; *n*, number; full mark, score 3; EGD, esophagogastroduodenoscopy; EUS, endoscopic ultrasonography; 95% CI, 95% confidence interval.

* *p* < 0.05.

### Endoscopic findings of the stomach

Using standard EGD and forward‐viewing radial EUS, 69 gastric lesions were detected in 69 participants as follows: 32 chronic gastritis, 23 polyps, four submucosal tumors, one carcinoma, three ulcers, five erosions, and one angiodysplasia (Table 4). The kappa statistic (κ) between standard EGD and forward‐viewing radial EUS for findings of chronic gastritis was as follows: κ = 1 for intestinal metaplasia and fold enlargement, κ = 0.956 for atrophy (95% CI, 0.871–1.042), κ = 0.900 for diffuse redness (95% CI, 0.696–1.104). The κ for RAC could not be calculated since the frontal part of the lesser curvature in the angulus in the retroflex view was not depicted on forward‐viewing radial EUS in half of the cases. The κ‐value for map‐like redness could not be calculated because of the unilateral finding (Table 5).

**TABLE 4:** Detected lesions of the stomach

**Endoscopic findings**	**Number of lesions**
Chronic gastritis	32
Polyps	23
Submucosal tumor	4
Carcinoma	1
Ulcer	3
Erosion	5
Angiodysplasia	1
Total	69

**TABLE 5:** Concordance rate between standard esophagogastroduodenoscopy (EGD) and forward‐viewing radial endoscopic ultrasonography (EUS) for findings of chronic gastritis in 32 cases

		**Standard EGD**	**Forward‐viewing radial EUS**	**κ**	**95% CI**
		**Number (%)**	**Number (%)**		
Atrophy	Close‐1	3 (9.4)	3 (9.4)	0.956	0.871–1.042
	Close‐2	14 (43.8)	13 (40.6)		
	Close‐3	3 (9.4)	3 (9.4)		
	Open‐1	9 (28.1)	10 (31.3)		
	Open‐2	1 (3.1)	1 (3.1)		
	Open‐3	2 (6.3)	2 (6.3)		
Intestinal metaplasia	Absent	29 (90.6)	29 (90.6)	1	
	Antrum	1 (3.1)	1 (3.1)		
	Corpus	2 (6.3)	2 (6.3)		
Fold enlargement	Absent	31 (96.9)	31 (96.9)	1	
	Present	1 (3.1)	1 (3.1)		
Diffuse redness	Absent	29 (90.6)	28 (87.5)	0.900	0.696–1.104
	Present	3 (9.4)	4 (12.5)		
RAC	Visible	0 (0)	0 (0)	N/A	
	Invisible	32 (100)	16 (50.0)		
	N/A	0 (0)	16 (50.0)		
Map‐like redness	Absent	32 (100)	32 (100)	N/A	
	Present	0 (0)	0 (0)		

Abbreviations: EGD, esophagogastroduodenoscopy; EUS, endoscopic ultrasonography; κ, kappa statistic; RAC, regular arrangement of collecting venules; N/A, not available; 95% CI, 95% confidence interval.

### Adverse event

No adverse event was observed in this study.

## DISCUSSION

This study described the depictability of 26 upper GI areas on both standard EGD and forward‐viewing radial EUS. Forward‐viewing radial EUS depicted 23 upper GI areas with satisfactory results, equivalent to the standard EGD. However, the depictability scores of the three angular areas with this scope were significantly lower than those of standard EGD. The rate of full‐mark scores in all 26 upper GI areas with forward‐viewing radial EUS, unfortunately, resulted in 20.3% and 95.7% in standard EGD. Considering the current situation, it is too early to introduce this forward‐viewing radial EUS for simultaneous investigation of the upper GI and pancreaticobiliary systems. If implemented, this EUS must be followed by a standard EGD when poor images are obtained in angular areas.

A previous study suggested that this forward‐viewing radial EUS was a useful screening modality for both the upper GI and pancreaticobiliary systems.^3^ The authors described that this EUS missed the lesser curvature of the angulus in the retroflex view by 37.8%.^3^ They advocated that this issue would be resolved in the short scope position.^3^ However, they did not evaluate both scopes in one session. This short scope position cannot always depict the frontal and inferior parts of the angulus. Invisible RAC in the angulus is one of the independent high‐risk endoscopic findings for gastric cancer.^12^ In our study, the frontal part of lesser curvature in angulus was not depicted on forward‐viewing radial EUS in half of the patients with chronic gastritis. Thus, the low depictability of the angulus on forward‐viewing radial EUS is an issue that needs to be addressed accordingly.

This forward‐viewing radial EUS still harbors the drawbacks described above; however, it potentially investigates the upper GI and pancreaticobiliary system in a single session, if the issue in the three angulus areas is overcome. As shown in Figure 1 and Table 1, EUS has a longer hard part of the tip compared to the standard EGD. Therefore, it is sometimes difficult to maintain an appropriate distance from the angular areas in the retroflex view. This issue can be solved if we cut the hard part slightly without loss of scanning performance for the pancreatic tail, and provide slight oblique viewing. When a dedicated scope is available, physicians can introduce this forward‐viewing radial EUS for the pancreaticobiliary system in the setting of endoscopic gastric cancer screening.^15^

Only 2.6%–9.0% of patients with pancreatic cancer present with resectable stage, and the 5‐year survival rate is 7.0%–15.0%.^16^ The recent 5‐year survival rate as reported in stage IV pancreatic cancer was 6.5%, however, that in stage IA was 54.1%.^17^ Although identifying pancreatic cancer as early as possible is essential, there are several challenges with its relatively low disease prevalence and diagnostic yield of existing modalities.^18^ Therefore, to date, screening of the asymptomatic adult population is not feasible.^18^ Recently, EUS is considered the most sensitive method for detecting early neoplasia in the pancreas.^19^, ^20^ EUS detects pancreatic tumors smaller than 20 mm with high accuracy of 87.7%–100%.^21^ However, EUS is performed under sedation, it is not a readily accessible imaging modality, and is highly dependent on the skill of the operator.^19^ Therefore, traditional EUS has a high threshold to be introduced solely as a pancreatic cancer screening modality. If the upper GI depictability of forward‐viewing radial EUS is equivalent to that of standard EGD, screening of the pancreaticobiliary system including the pancreas using this scope during upper GI screening would be more accessible.

There were mismatched findings for chronic gastritis between the two scopes. The reasons are as follows: first, the length of the scope tip influenced the depictability of RAC in angulus. Second, in this study, we performed a tight schedule of three examinations including standard EGD, endoscopic mode of forward‐viewing radial EUS, and EUS in one session. As described in Table 2, the duration time in each scope was short and dispersive. It was reported that slow EGD, which took over 420 s, improved the detection rate of lesions.^11^ We speculated that the length of scope tip and the observation time were the main reasons for this mismatch.

This study had several limitations. First, there was an observer bias in which investigators were educated beforehand to capture several images in all 26 upper GI areas. Second, the investigator and interpreter for each examination were the same. Third, the order of scope intubation was not randomized, and it was dependent on the operator's discretion. Fourth, this was a prospective study conducted at a single center. Fifth, EUS was performed only by expert hands or under expert supervision.

In conclusion, forward‐viewing radial EUS depicted upper GI areas equivalent to standard EGD, except for angular areas. EUS has the potential to simultaneously investigate the upper GI and pancreaticobiliary systems with further improvement.

## CONFLICT OF INTEREST

The authors declare they have no conflict of interest.

## FUNDING INFORMATION

This research received no specific grant from any funding agency in the public, commercial, or not‐for‐profit sectors.

## Data Availability

The data that support the findings of this study are available from the corresponding author upon reasonable request.
